# Halogen-Free Waterborne Polymeric Hybrid Coatings for Improved Fire Retardancy of Textiles

**DOI:** 10.3390/polym15234496

**Published:** 2023-11-23

**Authors:** Onur Yilmaz, Mehmet Kucuk, Raluca Nicoleta Darie-Nita, Catalina Natalia Cheaburu-Yilmaz

**Affiliations:** 1 Leather Engineering Department, Faculty of Engineering, Ege University, Bornova 35100, Izmir, Türkiye; onur.yilmaz@ege.edu.tr; 2 ACADEMICHEM Kimya ARGE San. Tic. Ltd. Şti, Ege University Technology Development Zone, Bornova 35100, Izmir, Türkiye; 3 Textile Engineering Department, Faculty of Engineering, Ege University, Bornova 35100, Izmir, Türkiye; mehmet.kucuk@ege.edu.tr; 4 Physical Chemistry of Polymers Department, “Petru Poni” Institute of Macromolecular Chemistry, Romanian Academy, 41A Grigore Ghica Voda Alley, 700487 Iasi, Romania; darier@icmpp.ro; 5 Biochemistry Division, Department of Chemistry, Faculty of Science, Dokuz Eylul University, Buca 35390, Izmir, Türkiye

**Keywords:** fire retardant, textile, hybrid coating, styrene-acrylic, composites, limit oxygen index

## Abstract

Wildfires are becoming more intense and more frequent, ravaging the habitations and ecosystems in their path. One solution to reducing the risk of damage to buildings and other structures during a fire event is the use of fire-retardant coatings that can stop or slow down the spread of flames, especially for textile materials. The present study focuses on the preparation and application of halogen-free boron/bentonite-based polymeric fire-retardant (FR) hybrid coating formulations for fabrics such as cotton (CO) and polyester (PE) fibers. For the preparation of FR composites, two types of boron derivatives, disodium octaborate and zinc borate, were used in combination with sodium bentonite. A styrene-acrylic copolymer was specifically synthesized and used as a coating binder for FR components to apply on fabrics. The properties of the synthesized copolymer and FR composites were characterized with a particle size analysis, FTIR spectroscopy, a dynamic mechanical thermal analysis (DMTA), and rheological measurements. The obtained hybrid composites based on styrene-acrylic copolymers and two different inorganic fillers were applied on cotton (CO) and polyester (PE) fabrics with a screen-printing technique, and the flame retardancy performance of the finished textile samples was investigated by means of flame spread and limit oxygen index (LOI) tests. The findings showed that the FR-composite-coated fabrics had higher LOI values and much decreased flame spread rates in comparison with uncoated ones. Among the boron derivatives, the composites prepared with disodium octaborate (FR-A) had much more pronounced LOI values and decreased flame spread behavior in comparison with the composite with zinc borate (FR-B). When compared to a commercial product, the FR-A composite, in conjunction with the specially synthesized polymer, demonstrated commendable fire retardancy performance and emerged as a promising candidate for a halogen-free waterborne fire-retardant coating for fabrics.

## 1. Introduction

Fire events have enormously increased in the last years due to global warming, accidents, the increased use of electrical equipment, etc. Therefore, fire protection in many fields has become important for our lives. The flammability characteristics of numerous textiles, particularly household textiles like curtains, carpets, and sheets, as well as upholstery materials (textile, leather, etc.) in transportation vehicles such as buses, trains, and planes, are of the utmost concern for fire safety. Correspondingly, the demand for flame-retardant textiles is on the rise [1]. To improve the flame retardancy of these materials, halogen, nitrogen, phosphorus, and minerals containing chemicals are usually incorporated into polymeric coatings [2,3,4]. Halogen- and formaldehyde-based fire retardants exhibit good anti-flame performance and are cost-effective; however, their application has become limited due to their toxicity and negative environmental impact [5,6]. Thus, there have been many attempts to improve the performance of halogen-free textile fire-retardant coatings.

Phosphorus- or phosphorus/nitrogen-based flame retardants such as tetrakis(hydroxymethyl) phosphonium salts and n-hydroxymethyl-3-(dimethoxyphosphono)propionamide have received attention in the last decade and have been used as halogen-free agents [7,8,9,10]. There have also been studies on the development of halogen-free fire retardants based on natural sources including proteins, tannins, phytic acid, chitosan, etc. [11,12,13,14]. Boron derivatives are also often used as flame retardants in various applications, including textiles, due to their ability to inhibit or delay the ignition and spread of flames. These derivatives work by releasing water vapor and other gasses when exposed to heat, which dilutes the flammable gasses produced during combustion. This dilution suppresses the combustion process and reduces the likelihood of a fire spreading. In textile applications, boron derivatives are incorporated into fabrics and fibers to enhance their flame resistance. Some commonly used boron-based flame retardants for textiles include boric acid, borax, boron nitride, and boron complexes [15,16,17,18]. However, the sole use of these kinds of flame retardants in the treatment of cotton fabrics usually exhibits insufficient retardant behavior, or they have to be used in high amounts for an effective flame-retardant activity.

One way to improve fire retardancy is the use of hybrid flame-retardant systems that combine different kinds of agents such as organic and inorganic compounds. In many studies, flame retardancy and the thermal stability of materials like polymers and textiles have been improved by this cooperative effect. In this system, the inorganic content contributes to thermal stability, whereas the organic part provides higher char formation [19,20,21,22]. In recent examples of this approach, super-hydrophobic flame-retardant coatings based on phosphoramidate/siloxane (DPTES) were prepared and applied to the surface of cotton with sol–gel technology [23]. In another study, flame-retardant and hydrophobic cotton textiles through a dual-dip-coating technique were prepared using complex dual solutions (CSs) consisting of sustainable/renewable materials such as chitosan, urea, phytic acid, and silicon-containing substances, i.e., tetraethyl orthosilicate and hexamethyldisiloxane which were rich in silica (Si), phosphate (P), and nitrogen (N) [24]. An organic–inorganic hybrid coating to improve the fire safety and antibacterial properties of polyester/cotton blend fabrics was developed [25]. The authors constructed the hybrid coating via the in situ growth of zirconium phosphate nanosheets and the preparation of a polyelectrolyte complex with phytic acid and poly(hexamethylene guanidine hydrochloride) via the spraying method. In another approach, a flame-resistant gel/textile composite was fabricated with a simple casting method [26]. For this purpose, acrylamide (AAM) and SiO_2_ were added into an aqueous clay dispersion and further mixed with polyethylene glycol diacrylate and polyethylene glycol methacrylate. Potassium persulfate and TEMED (N,N,N′,N′-tetramethylethylenediamine) were added, and the pre-gel solution was immediately transferred into a mold with a viscose non-woven fabric at the base and polymerized. After polymerization at 30 °C for 1 h, a Gel/Tex composite (FR-GT) was successfully obtained. A hybrid sol using silica sol, a coupling agent of γ-methacryloxypropyltrimethoxysilane, and flame-retardant sodium octaborate tetrahydrate (DOT) were prepared and then used for finishing the surface of the cotton fabric [27]. These studies have unveiled the enhanced fire-retardant efficacy of such hybrid coatings. The mentioned fire-retardant components are typically applied with a polymer dispersion coating to affix them onto the textiles and enhance their thermal stability. Furthermore, a judicious selection of the polymeric binder may also enhance the overall system’s performance [28,29,30].

Styrene acrylic emulsions are widely used as coatings for textiles, train cars, ship interiors, buildings, architectural coatings, adhesives, paper coatings, etc. Coatings based on styrene acrylate emulsions display excellent UV stability, resistance to water, detergents, and alkalis, and they have low-volatile organic compounds (VOCs) [31,32]. Although they cannot be utilized alone as fire-retardant materials, they can be used as binders for fire-retardant additives to different substrates with good coating performances [33,34,35,36,37,38].

The present study focused on the innovative preparation and application of halogen-free hybrid fire-retardant compositions based on a styrene-acrylic copolymer dispersion, sodium bentonite, and two different boron derivatives: zinc borate and disodium octaborate. The copolymer used as a binder for the fire-retardant composition was specifically synthesized to enhance the coating performance of the fire-retardant composites. The properties of the copolymer, coating composition, and flame-retardant performance of the coated textile materials were evaluated and are discussed in this paper.

## 2. Materials and Methods

### 2.1. Materials

Butyl acrylate (BA, ≥99.0%), methyl methacrylate (MMA, ≥98.5%), styrene (St, ≥97%), methacrylic acid (MAA, ≥99.0%), and acrylonitrile (ACN, ≥99.0%) were purchased from Sigma-Aldrich, Schnelldorf, Germany and used as monomers in the emulsion copolymerization for the styrene-acrylic copolymer synthesis. An emulsifier system containing Disponil SLS 101, Texapon^®^ P, and Disponil FES77 were products of BASF, Ludwigshafen, Germany, and used for the stabilization of the emulsion. Sodium bicarbonate (NaHCO_3,_ ≥99.0%, Merck, Darmstadt, Germany) and ammonium persulfate (APS, ≥98.0%, Merck, Darmstadt, Germany) were used as the buffer agent and initiator, respectively. All chemicals were used as received, without any further purification. Ultrapure water was used as a solvent to perform the reactions. The syntheses took place in a four-necked round bottom 250 mL glass reactor equipped with a condenser, dropping funnels, and a mechanical mixer. An IKA (Staufen, Germany) temperature control probe heater with an oil bath was used for the reactions and formulation preparation.

For the preparation of the flame-retardant composition, sodium bentonite (Sigma-Aldrich), zinc borate 3.5 hydrate (2ZnO·3B_2_O_2_·3.5H_2_O, Etimaden, Ankara, Türkiye), and disodium octaborate tetrahydrate (Na_2_B_8_O_13_·4H_2_O, Etimaden, Ankara, Türkiye) were used as inorganic fillers.

Textile fabrics based on cotton and polyester were used to apply the flame-retardant composition. A commonly preferred commercial waterborne fire-retardant product (FR-C) for textiles with a synergic effect (polymer dispersion with antimony trioxide and chlorinated paraffin) was used as a comparison to evaluate the final flame-retardant performance.

### 2.2. Synthesis of Copolymer Emulsion

**Scheme 1:**
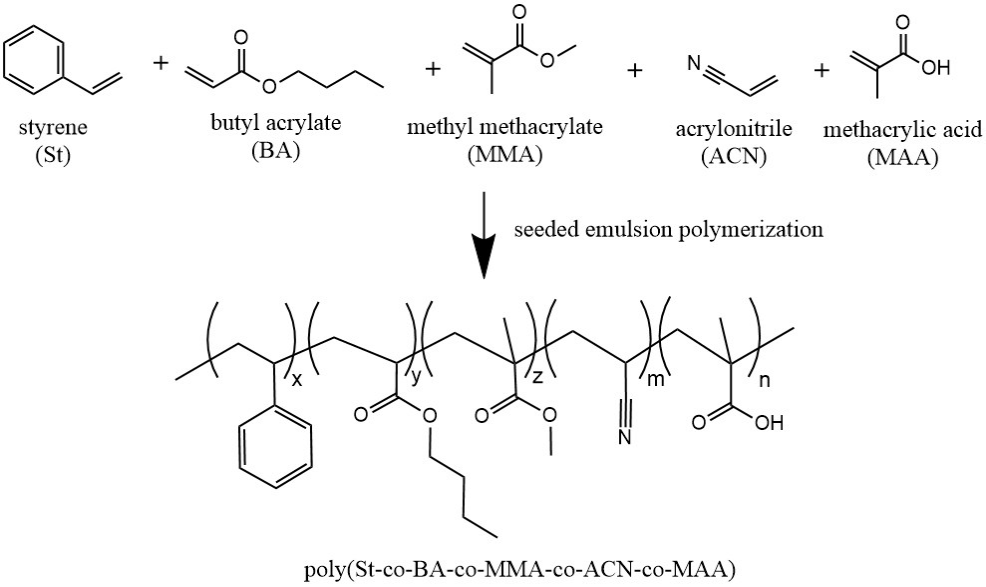
The synthesis route of the emulsion polymer used as a coating binder for FR composites.

The copolymer dispersion used as a binder for the formulations to coat the textiles was synthesized via the seeded emulsion polymerization technique. For the synthesis, the emulsifiers and NaHCO_3_ were dissolved in water at room temperature in the reactor and mixed for 15 min. A total of 5 wt.% of the total monomer mixture composed of St, MMA, BA, MAA, and ACN was added in the reactor and mixed well. The temperature was raised to 75 °C, and a portion of the APS solution was added to initiate the radical polymerization to form the seed. After 15 min, the remaining monomer mixture and APS solution were continuously fed in the reaction for 3 h in separate funnels. After all transfers were completed, the system was maintained for an additional 90 min to complete the reaction. The synthesis route is given in Scheme 1. The conversion of the reaction was periodically checked by precipitating the aliquots in acetone, filtering, drying, and weighing the dried polymer. The final conversion of the reaction was obtained above 99%. Subsequent to cooling the system, a coagulum-free emulsion, blueish and transparent, with a solid content of 30 ± 1.0 wt.% was obtained. The final pH of the emulsion was adjusted to 7.0 ± 0.5 by using concentrated triethanolamine (Merck, ≥99.0%).

### 2.3. Preparation of FR Compositions for the Textile Coating

The formulations applied on the textile samples to function as fire-retardant coatings were prepared with the blending technique using a mechanical stirring device at 1000 rpm. Two compositions were separately prepared (FR-A and FR-B) based on the type of boron derivatives. Firstly, the selected amount of the copolymer dispersion was placed in a 500 mL beaker and heated to 45 °C while mixing at 500 rpm. Sodium bentonite was first dispersed in 10 mL of water, mixed at a high speed (1000 rpm), and ultra-sonicated for 15 min. The viscous bentonite solution was carefully added into the copolymer dispersion during mixing. Subsequently, disodium octaborate or zinc borate solutions were slowly added into the composition. Finally, two fire-retardant (FR) compositions (FR-A: with disodium octaborate; FR-B: with zinc borate) were obtained after mixing for 2 h at 1000 rpm. The inorganic filler content of the final FR composites was ≤25 wt.%.

### 2.4. Application of FR Compositions to Fabrics

**Figure 1:**
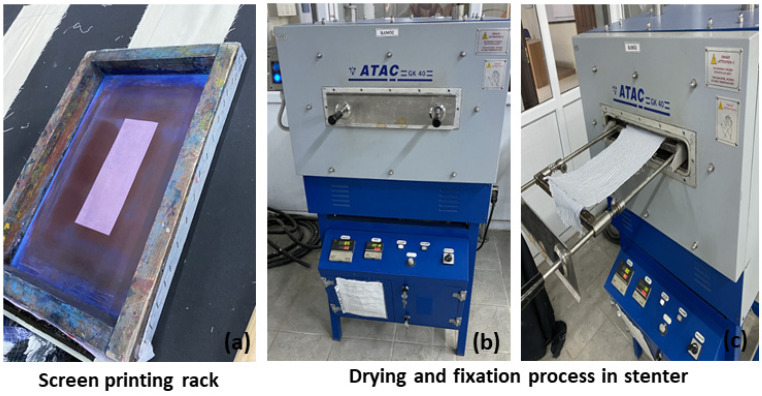
Screen-printing rack (**a**) and drying and fixation process in stenter (**b**,**c**).

The previously prepared two FR compositions (FR-A and FR-B), as well as a commercial FR product (FR-C), were applied to cotton and polyester fabrics by coating the surface using the screen-printing technique (Figure 1). Compositions were directly applied without further dilution. Prior to tests, the fabrics were conditioned at a 20 ± 2 °C temperature and 65 ± 2% relative humidity for 24 h. After the application step, the fabrics were transferred to a stenter machine for drying at 100 °C for 3 min (Figure 1b,c). After drying, all the samples were subjected to the fixation process carried out in the stenter at 150 °C for 5 min. The application intensity was calculated in terms of the weight of the coating material per unit area of the fabric by weighing the fabric (with measured area) before and after the application. The calculation showed that the application intensity of the compositions on CO fabric was 159 ± 6 g/m^2^, and for PE fabric, it was 66 g/m^2^. The thickness of the untreated CO fabric was 0.62 ± 0.015 mm, and for the PE fabric, it was 0.28 ± 0.010 mm.

### 2.5. Characterization Methods

The particle size distribution and polydispersity index of the synthesized copolymer dispersion were evaluated using a NanoZS Zetasizer instrument (Malvern Instruments, Worcestershire, UK).

The structure of the copolymer was confirmed with FTIR analysis recorded with a Bruker VERTEX 70 spectrometer (Bruker, Billerica, MA, USA). The analysis was performed on polymer films obtained by casting the emulsions on Petri dishes and subsequent drying at room temperature. The spectrum was obtained after 5 scans between 4000–600 cm^−1^ using reflection on a diamond crystal with an angle of 45° and a resolution of 4 cm^−1^.

Dynamic mechanical measurements were performed on the coating compositions using an Anton Paar MCR 301 Dynamic Mechanical Analyzer (Graz, Austria) equipped with CTD450, in the temperature range from −55 to 190 °C, under a N_2_ atmosphere, with a heating rate of 3 °C/min. A film clamp was used and a heating rate of 3 °C/min was employed to ensure the thermal equilibrium of the sample with the instrument. The measurements were carried out in the extension mode at 1 Hz frequency. The variation of the viscoelastic parameters, such as the storage modulus E′, loss modulus E″ vs. temperature (T), and the value of the tan δ (tan δ = E′/E″), were recorded for all temperature ranges.

The rheological properties of the FR compositions were measured with an Anton Paar 301 Rheometer (Graz, Austria) device using a cone/plate geometry measuring system with an angle of 1.003° at 25 °C. Flow behavior (Newtonian or non-Newtonian) was tested with the rotational controlled shear rate condition (CSR), where the viscosity (*η*) of the samples was measured as a function of the increasing shear rate (*γ* = 0.1–1000 s^−1^). The viscoelastic behavior of the compositions, such as the storage (G′) and loss moduli (G″) of the compositions, was measured as a function of angular frequency (ω = 0.1–1000 s^−1^) using oscillatory tests. To perform the frequency sweep tests, the linear viscoelastic range of the samples (LVE) was obtained from amplitude sweep tests (with a strain amplitude between 0.01 and 500%) using a constant angular frequency ω = 10 s^−1^.

The flame spread properties of the vertically oriented coated fabrics were investigated with the standard test for limited flame spread [39]. All the flame spread tests were performed in three replicates for the same sample.

The flame-retardant behavior of the samples was also evaluated by determining the limiting oxygen index (LOI) according to the standard oxygen index test [40]. The LOI value indicated the volume fraction concentration of oxygen just supporting combustion in the mixture of oxygen and nitrogen gasses. The fabric samples cut in the dimensions of 125 × 6.5 × 3.2 mm were tested at least five times for each sample, and the average values are presented.

## 3. Results and Discussion

### 3.1. Copolymer Properties

**Figure 2:**
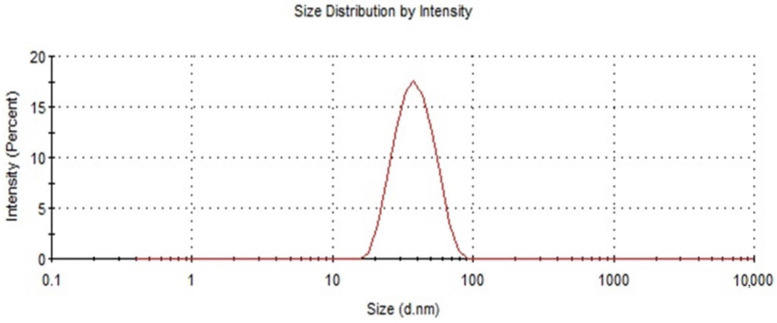
Particle size distribution curve of the copolymer dispersion.

A copolymer dispersion composed of St, MMA, BA, MAA, and ACN monomers with a ratio of 28.2/3.5/57.0/3.3/8.0 by weight, respectively, was synthesized to be used as a coating binder in fire-retardant compositions. The monomers St, MMA, and ACN were selected due to their ability to improve the mechanical properties and thermal stability of the copolymer composition. BA was selected to increase the softness to that suitable for flexible textiles, while MAA was used to enhance the stability and adhesion abilities of the coating. Seeded emulsion polymerization was carried out for the synthesis of copolymer to facilitate a good particle stability since the ratios of hydrophobic monomers (St and BA) were high in the composition. At the end of the reaction, a stable, transparent-like, coagulum-free copolymer dispersion was obtained. The particle size distribution of the copolymer dispersion obtained from a Zetasizer is plotted in Figure 2. From the analysis, the average particle size diameter of the latex particle was found to be 39 nm with a polydispersity index value (PDI) of 0.07. The result showed that the copolymer had a very fine particle size and homogenous size distribution. The small particle size of the dispersion was especially adjusted to increase the surface area of the particles to facilitate a better binding capacity for inorganic fillers and pigments as well as to improve the penetration and adhesion to the fabric.

**Figure 3:**
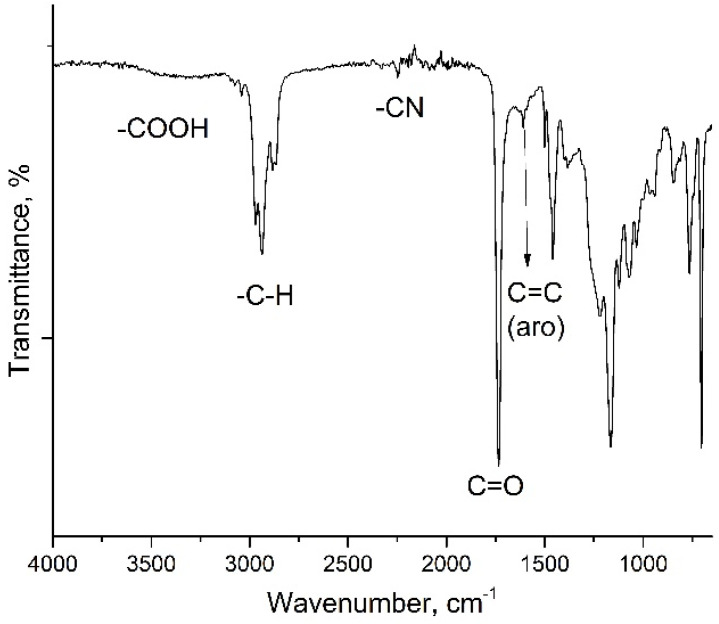
FTIR spectrum of the copolymer film.

The chemical characterization of the copolymer binder was performed via FTIR spectroscopy, and the spectrum is given in Figure 3. From the spectrum, the main characteristic absorption peaks of the copolymer were observed from 3130 to 3600 cm^−1^ and 2800 to 3000 cm^−1^, and at 2247, 1734, 1608, 1459, 1163, 763, and 702 cm^−1^, respectively, which could be attributed to the –OH stretching of COOH groups, the asymmetrical and symmetrical -CH group stretching of CH_2_ and -CH_3_, the stretching vibration of the -CN group, -C=O stretching vibration, the C=C stretching of the aromatic ring, -CH_2_ and -CH_3_ deformation, C-O-C stretching, or the -CH bending of the aromatic ring, respectively. The IR interpretation of the spectrum was performed using the available accepted FTIR spectra databases and the literature [28,41]. The absence of the vibration peaks around 1608–1634 cm^−1^ assigned to the vinyl groups of acrylates for both latexes marked the success of the polymerization.

### 3.2. Properties of FR Compositions

#### 3.2.1. Thermo-Mechanical Properties

**Figure 4:**
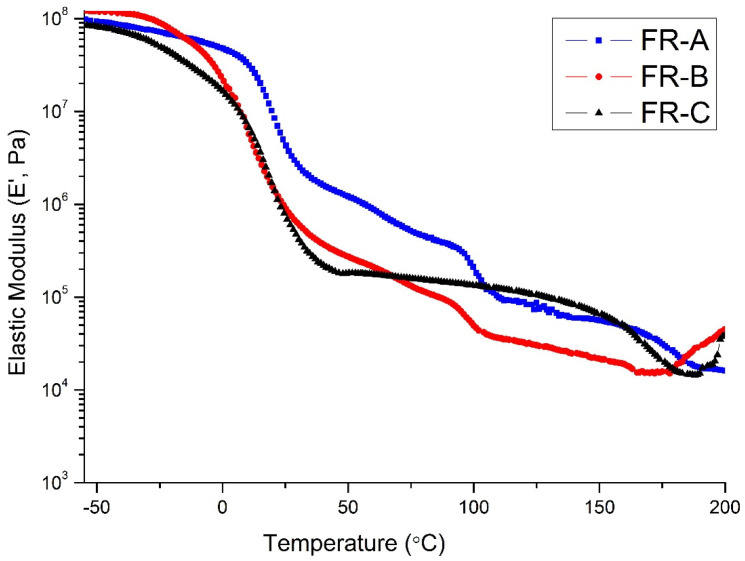
Elastic moduli curves (E′) of the FR composites as a function of temperature.

**Figure 5:**
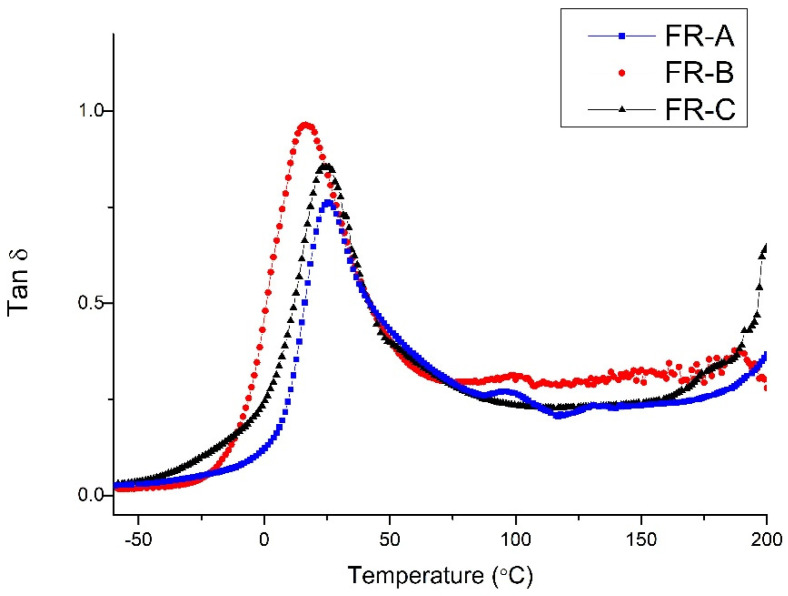
Tan δ curves of the FR composites.

Dynamic mechanical thermal analysis (DMTA) was conducted in order to examine the viscoelastic properties of the compositions in the temperature range. In Figure 4, the change of the elastic modulus versus temperature, and in Figure 5, the Tan δ curves, are presented for the two newly developed FR compositions and the commercial product. The curves revealed for FR-B a relaxation region between −25 °C and 60 °C due to the orientation of the molecular chains and a peak at 15 °C indicating the glass transition temperature (T_g_). The commercial product exhibited a continuous relaxation behavior up to 45 °C with a T_g_ peak at 24° C, followed by a rubbery region until 160 °C and a melting process afterward. The other FR-A composite had a relaxation region between −5 °C and 45 °C with a T_g_ peak at 24 °C. Both FR composites (FR-A and FR-B) showed a second small-phase changing behavior at around 97 °C, which could have been possible due to the relaxation of partially confined chains between the inorganic crystalline structures of bentonite/boron in the composites. After evaluating the elastic modulus values for the testing temperature range, the composite FR-A showed a much higher modulus than the other composites, possibly due to a better dispersion and the interaction of inorganic fillers within the polymer matrix. The measure of the storage modulus (and also the loss modulus and tan delta) as a function of temperature change in DMA tests offered valuable information regarding different transitions (depending on the material chemistry), with insights into the material’s thermal and mechanical properties, including the glass transition temperature, which could then be used as a quality control metric that could predict the performance of the respective material. A high elastic modulus in DMA tests refers to the enhanced stiffness of the respective material and its higher energy storage capability and internal properties related to thermal stability [42]. Usually, the degree of interfacial adhesion at the boundary of the polymer and inorganic filler as well as the particle size and the distribution of the filler affect not only the mechanical properties of the composites but also the fire-retardant properties. It is well established in the field of fire retardancy that specific interactions between polymers, fire-retardants, and other components have a significant impact on the flammability of the polymer composite [43,44].

#### 3.2.2. Flow Behavior of the FR Composites

The flow behavior and viscoelastic properties of the FR products are important to understand, as well as their performance during application to fabrics, their resistance to shear forces, and their stability and shelf-life. For this purpose, rheological tests were performed on the prepared FR composites and the commercial product for comparison.

**Figure 6:**
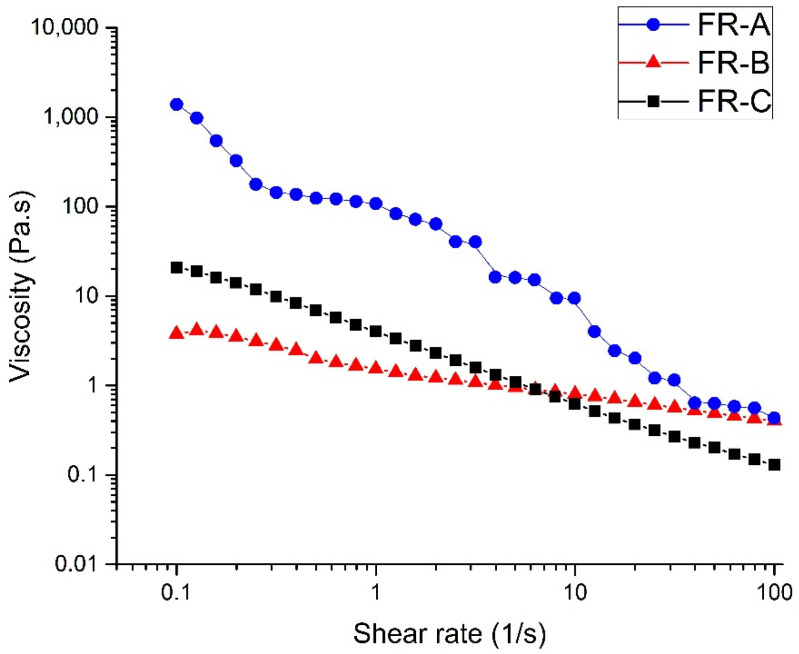
Shear viscosity curves of the FR composites.

The flow behavior of the composites is shown in Figure 6 where the viscosity changed as a function of shear rate. When the low shear viscosities of the samples are compared, one can observe that FR-A displayed the highest viscosity possible due to the corresponding increase in the volume-specific surface (the ratio between the droplet surface and droplet volume), and the resulting increase in the interactions between the droplets led to higher values of the flow resistance (viscosity). The composite FR-B showed decreased viscosity values compared with the commercial product at low shear rates. Moreover, all the FR compositions revealed a decrease in viscosity with increasing shear rates, exhibiting a shear-thinning behavior. Usually, polymers have a tendency to entangle with their neighboring macromolecules in their three-dimensional network at a “rest” state (low shear rates). But, during the shear process, the molecules are usually oriented in the shear direction by entangling to a certain extent, which lowers their flow resistance [45]. Viscosity is an important factor for coating applications in every case, whether the composition is printed, sprayed, rolled, or curtain-coated. If the viscosity is not proper, serious problems like flowing, dripping, or insufficient spreading and thick coating layers may occur [46]. Therefore, a shear-thinning behavior is desired for coating applications to facilitate a uniform transfer of the composition to the substrate and a relatively high viscosity at low shear rates to avoid flowing and dripping from the surface during settling and drying. Taking into account the flow behavior of the FR compositions, FR-A especially was found to be proper for roll-coat, spraying, and padding applications to textile materials.

**Figure 7:**
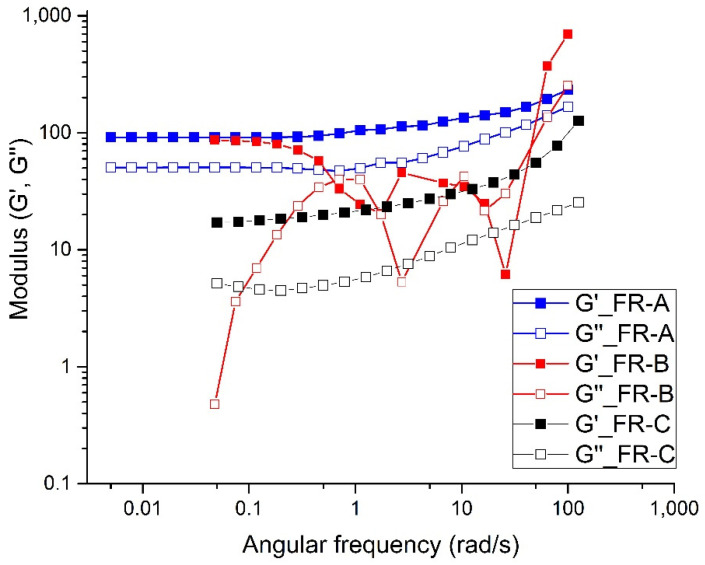
Storage (G′) and loss (G″) moduli curves of the FR composites.

Figure 7 displays the storage (G′) and loss moduli (G″) of the composites as functions of the angular frequency under a constant amplitude strain (LVE range). The results showed that FR-A and FR-C had a higher G′ than G″ for all frequency ranges, which indicated that the elastic behavior was more pronounced than the viscous behavior. In stable emulsions, dispersions’ or gels’ intermolecular interaction forces form three-dimensional networks and thus show G′ > G″ among all frequency ranges with a slight increase in the slope at higher frequencies [47]. Moreover, FR-A showed a higher storage modulus for the entire frequency range in comparison with the other composites, revealing a good compatibility with the copolymer matrix and fillers with increased intermolecular interactions that resulted in higher moduli values. This kind of improved elastic network led to an increase in the emulsion stability and shelf-life of the products. On the other hand, the composition FR-B showed multiple crossovers between G′ and G″ curves and a decreasing trend in the storage modulus with increasing shear rates. This behavior could have been due to insufficient compatibility and interactions between inorganic fillers (i.e., zinc borate) and the polymer matrix leading to the decreased uniformity and stability of the emulsion.

#### 3.2.3. Fire-Retardant Properties

The fire-retardant properties of the prepared composites were investigated with flame spread and LOI tests using official standard methods. In the testing experiments, all three FR samples were applied on pure cotton (CO) and polyester (PE) fabrics and tested after drying and fixing processes.

**Figure 8:**
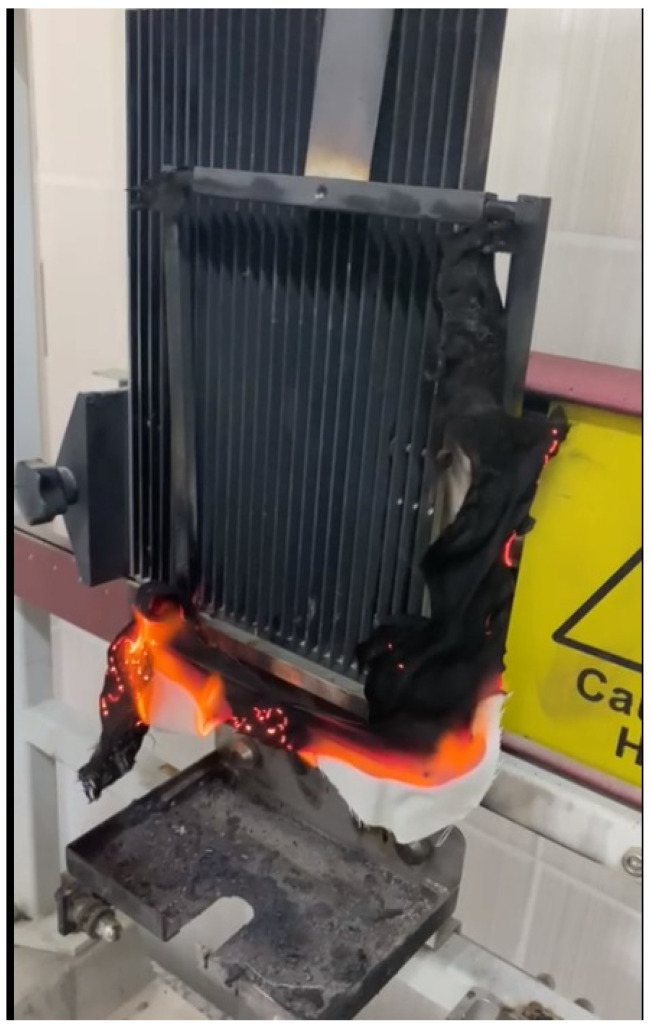
The images taken during the vertical flame spread tests performed on uncoated CO fabric.

**Figure 9:**
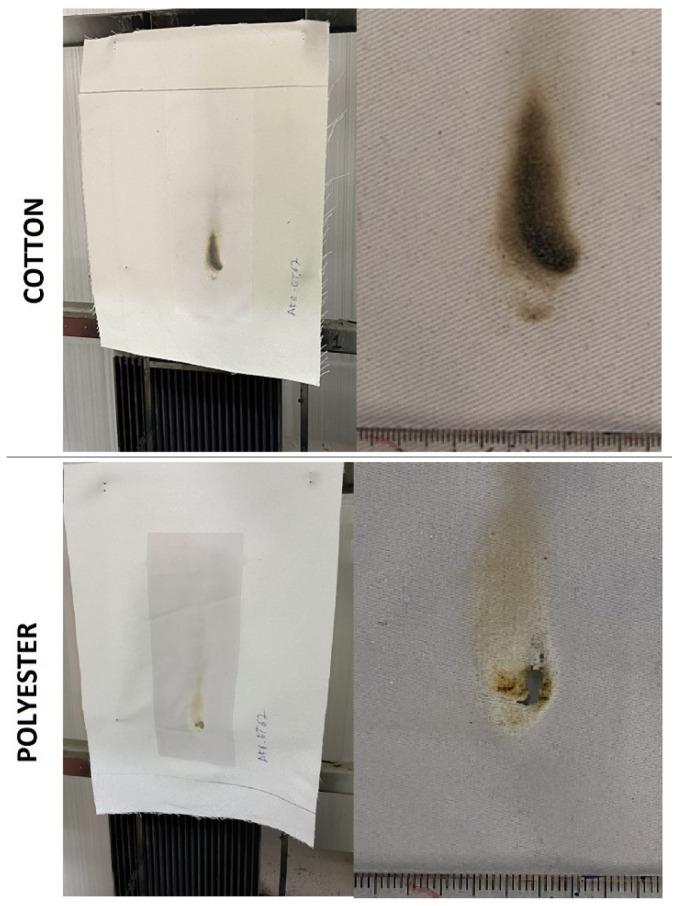
The images taken during the vertical flame spread tests performed on FR-A-coated CO (**above**) and PE (**below**) fabrics.

In the flame spread tests, all the coated fabrics with FR composites showed an increased performance in flame spread since the uncoated fabrics were completely burned at the end of the test (Figure 8). The CO fabric coated with FR-A did not show significant flaming or flame spread within the fabric. Moreover, no melting or melt dripping were observed in any of the samples. After the flame source was removed, the smoldering time was less than 2 s. No hole was observed in the sample after the experiment (Figure 9 above). Similarly, for the PE fabric coated with FR-A, no flame spreading behavior was observed throughout the fabric. After the flame source was removed, the duration of flaming and smoldering was less than 2 s. A small hole formation was observed in the PE sample (Figure 9 below).

**Figure 10:**
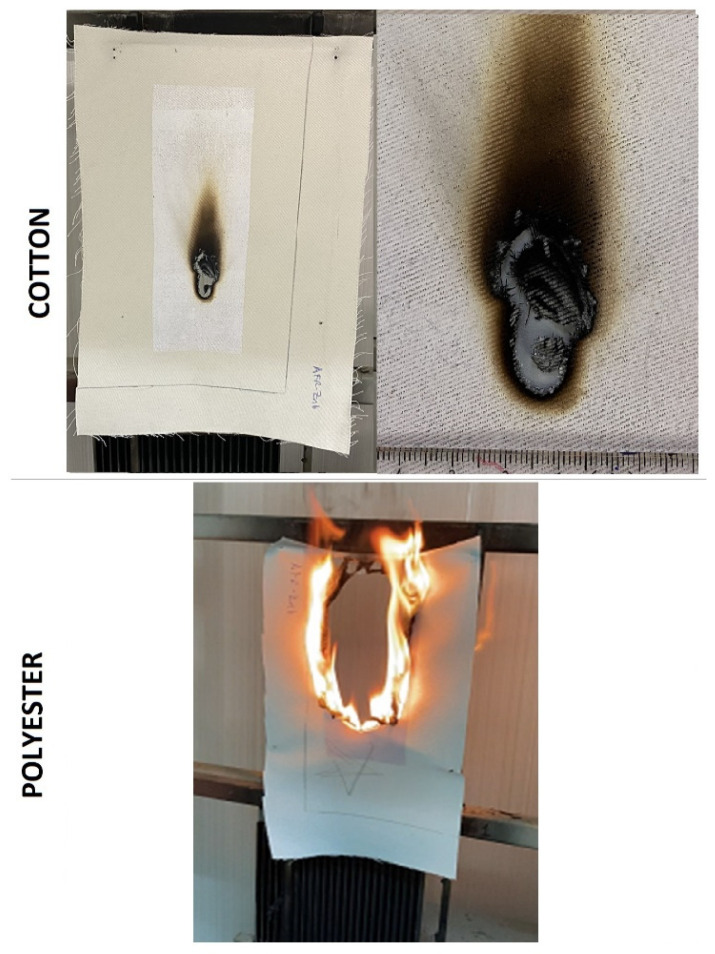
The images taken during the vertical flame spread tests performed on FR-B-coated CO (**above**) and PE (**below**) fabrics.

For the CO fabrics coated with FR-B, the flame did not reach the edges of the textile sample throughout the test. Furthermore, no melting or melt dripping were observed. However, after removal of the flame source, the duration of flaming and smoldering was longer than 2 s, and a hole was observed in the CO sample (Figure 10 above). The PE fabric coated with FR-B was burnt until the end of the test; however, the spread of the flame was slower than that of the untreated PE fabric (Figure 10 below).

**Figure 11:**
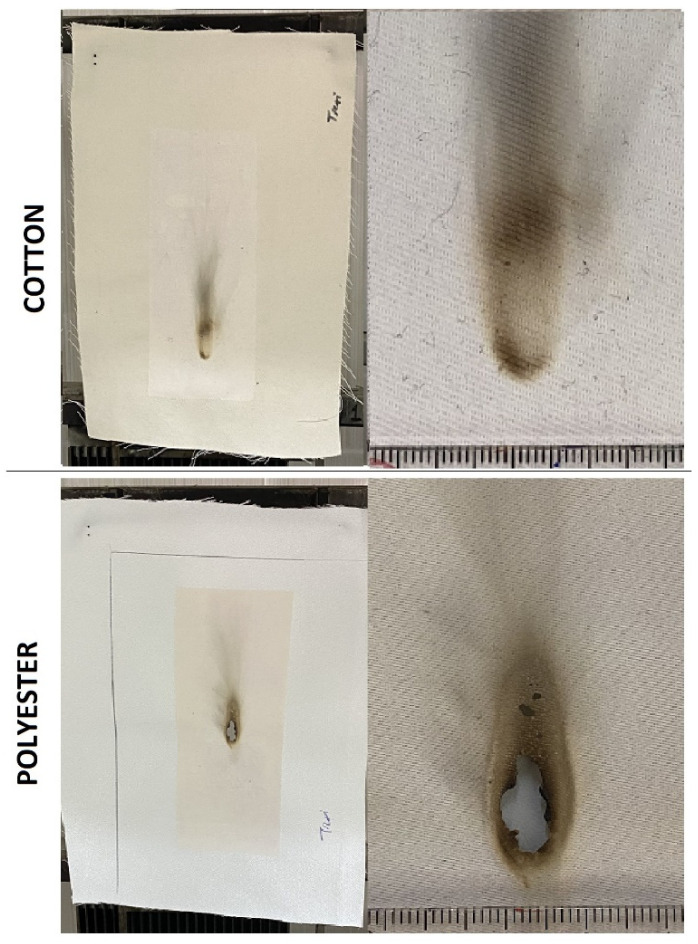
The images taken during the vertical flame spread tests performed on FR-C-coated CO (**above**) and PE (**below**) fabrics.

The flame spread test was also applied to the CO and PE samples coated with a commercial FR product (FR-C). In the CO fabric, no flame spreading, melting, or melt dripping were observed. After the flame source was removed, the flaming and smoldering time were also less than 2 s, and no hole was observed in the fabric. For the PE fabric coated with FR-C, no flame spread to the edges of the fabric; however, a melting behavior on the ignition point was observed in the sample. Following the removal of the flame source, the duration of flaming and smoldering was less than 2 s, and a hole formation was observed in the fabric (Figure 11).

The fire-retardant behavior of the composites was also investigated with LOI tests applied to coated CO and PE fabrics. LOI denotes the minimum concentration (vol.%) of O_2_ in a mixture of O_2_ and N_2_ that only sustain the flaming combustion of a material in a candle-like manner. Textile materials having LOI values up to 21 vol.% rapidly burn, while they slowly burn when the LOI is between 21 and 25 vol.%, whereas LOI values beyond 26 vol.% pose some flame-retardant properties [48]. The results obtained from the LOI test are given in Table 1. It was shown that the LOI values of uncoated CO (18.5) and PE (19.5) fabrics significantly increased for both fabrics coated with FR-A and FR-C and slightly increased for FR-B-coated fabrics. Moreover, the LOI value of the FR-A-coated CO fabric was found to be very high, with a value of 39.2, much higher than that of the commercial product, indicating its efficient flame retardancy.

## 4. Conclusions

The investigation into halogen-free waterborne polymeric hybrid coatings aiming to enhance the fire retardancy of textiles yielded promising results. The incorporation of boron derivatives, namely disodium octaborate and zinc borate, along with sodium bentonite, demonstrated a good cooperative effect in improving the fire-retardant properties of the coated cotton and polyester fabrics.

One of the key components of this research was the synthesized styrene-acrylic copolymer with a fine particle size incorporated into the formulation, which was found to improve the coating performance of the FR components within the fabric. The thermomechanical and rheological properties of the FR composites together with the fire-retardant properties assessed the importance of the interaction and compatibility of the polymer with inorganic fillers on desired properties. The flame spread test exhibited that the coated fabrics with FR composites did not show significant flaming within the fabric, and the smoldering was less than 2 seconds in most cases except for the PE fabric coated with FR-B. The LOI test also showed a remarkable increase in the fire-retardant performance for the FR-composite-coated fabrics. The LOI value of 18.3 for the uncoated CO fabrics increased to 22.6 with FR-B and 39.2 with FR-A. For the PE fabric, the LOI value of 19.5 improved to 19.7 and 26.4 after treatment with FR-B and FR-A, respectively. The results demonstrated that among the two boron derivatives, disodium octaborate (FR-A) revealed a standout FR performance, yielding superior LOI and flame spread results, especially when applied to cotton fabrics. By juxtaposing the outcomes with a commercial product (FR-C), the hybrid formulation involving the designed copolymer proved to be a potential compelling contender in the domain of halogen-free fire-retardant coatings for flexible materials such as textiles, leather, fibers, etc.

## Figures and Tables

**Table 1:** LOI values of the uncoated and coated fabrics.

Coating Composition	Fabric Type	LOI Value (vol. %)
FR-A	CO	39.2
FR-A	PE	26.4
FR-B	CO	22.6
FR-B	PE	19.7
FR-C	CO	28.0
FR-C	PE	28.8
None	CO	18.3
None	PE	19.5

## Data Availability

The data that support the findings of this study are available on request from the corresponding author.
